# Metabolic power, volatile compounds, and flavor marker formation mechanisms of three fungi isolated from Fengxiangxing Daqu

**DOI:** 10.3389/fmicb.2025.1593638

**Published:** 2025-05-09

**Authors:** Dan Cao, Jiali Lv, Cui Liu, Chengyong Jin, Yongli Zhang, Yuhang Zhang, Wen Zhang

**Affiliations:** ^1^School of Food Science and Engineering, Shaanxi University of Science and Technology, Xi’an, China; ^2^Shaanxi Xifeng Liquor Co., Ltd., Baoji, China

**Keywords:** Fengxiangxing Daqu, *Aspergillus chevalieri*, *Saccharomycopsis fibuligera*, *Thermoascus aurantiacus*, flavor markers, volatile compounds

## Abstract

**Introduction:**

Chinese Daqu is a naturally formed microbial complex, which is the fermenter of Chinese Baijiu. The microorganisms in Chinese Daqu determine the flavor of Chinese Baijiu.

**Methods:**

Based on PacBio SMRT ITS sequencing technology, fungal genome de novo sequencing, and Headspace Solid-Phase Micro-extraction/Gas Chromatography-Mass Spectrometry analyzed and identified three fungi, their abundance, volatile compounds, and flavor markers in Daqu.

**Results:**

*Aspergillus chevalieri*, *Saccharomycopsis fibuligera*, and *Thermoascus aurantiacus* were the three most abundant fungi, with relative abundances of 5.702, 31.686, and 62.256%, respectively. *Aspergillus chevalieri* and *Saccharomycopsis fibuligera* were associated with volatile markers of chained terpene alcohols and lactones, with linalool and γ-nonanolactone identified as their flavor markers, respectively. *Thermoascus aurantiacus* produced the highest variety and content of pyrazine. According to the functional annotation and metabolic relationship of the whole genomes of *Aspergillus chevalieri* and *Saccharomycopsis fibuligera* in COG, KEGG and other databases, the formation mechanism and possible metabolic pathways of linalool and γ-nonanolactone were elucidated. The results of the study revealed the different contributions of the three dominant fungi to the volatile compounds of Daqu, can provide ideas for the study of the contribution of other microorganisms to the volatile compounds of Daqu and the mechanism. It can provide a basis for the precise adjustment of the flavor of Daqu, and then the precise modification of the flavor of Baijiu.

## 1 Introduction

Chinese Baijiu has a production history spanning more than 2,000 years and accounts for approximately one-third of the global distilled spirits market (Tu et al., 2022; Wang et al., 2022). Chinese liquor is categorized into four major aroma types: Jiangxiang, Nongxiang, Qingxiang, and Fengxiang (Ren et al., 2024). Fengxiangxing, a distilled liquor exclusive to China with a long history and distinctive flavor, is also characterized by its unique brewing process and storage practices (Liu et al., 2022a).

Fengxiangxing Baijiu is a distilled spirit made primarily from barley, wheat, peas, sorghum, and other grains. It is prepared using Fengxiangxing Daqu as the saccharification and fermentation agent through saccharification, fermentation, and distillation (Tu et al., 2022). The formation of Fengxiangxing Baijiu can primarily be attributed to the unique geographical, environmental, raw materials, and process conditions determined by Fengxiangxing Daqu, with its unique flora serving as the core factor (Jia et al., 2022). Currently, the evaluation of Daqu quality primarily focuses on sensory characteristics and physicochemical properties. However, since the microbial flora in Daqu is its core component, establishing a fungal flora evaluation system could be instrumental in ensuring the stability of Baijiu aroma. By detecting and controlling the microbial flora, it is possible to maintain consistency in flavor. Exploring the characteristics and mechanisms of core flora within Daqu can further unveil the intrinsic nature of various Baijiu flavors, facilitating the transformation of Chinese Baijiu from traditional practices to intelligent processes. However, despite its importance, research on the characterization of core functional flora in Daqu and the mechanisms behind the flavor compounds they produce remain limited.

Currently, research on the fungal flora in Fengxiangxing Daqu primarily focuses on the diversity of the flora and its correlation with the genus-level microorganisms, physicochemical properties, and volatile compounds of Daqu. Studies have identified the five most abundant fungal genera as *Thermoascus*, *Saccharomycopsis*, *Aspergillus*, *Wickerhamomyces*, and *Kazachstania* (Zhang et al., 2023; Zhang et al., 2022a; Zhang et al., 2022b). These genera had different correlations with the liquefaction power, saccharification power, fermentation power, esterification power, and volatile compounds in Daqu. However, genus-level analyses were unable to identify the specific species responsible for these effects. Some studies on fungal species revealed the top five fungal species in order of abundance were *Thermoascus aurantiacus, Saccharomycopsis fibuligera, Aspergillus chevalieri, Wickerhamomyces anomalus,* and *Kazachstania humilis*. Among them, *Aspergillus chevalieri* and *Thermoascus aurantiacus* positively correlated with liquefaction power, saccharification power, and esterification power. In contrast, *Saccharomycopsis fibuligera* and *Wickerhamomyces anomalus* showed negative correlations with liquefaction, saccharification, and esterification power. Additionally, *Saccharomycopsis fibuligera* showed a positive correlation with the fermentation power of Daqu (Cao et al., 2024a; Cao et al., 2024b). These fungal species also exhibited different correlations with various volatile compounds in Daqu, including alcohols, aldehydes, acids, esters, ketones, phenols, nitrogen-containing compounds, sulfur-containing compounds, terpenes, lactones, furans, and aromatics (Cao et al., 2024a; Cao et al., 2024b; Chu et al., 2024). However, correlation analysis between the fungal flora and the physicochemical properties and volatile compounds of Daqu, based on microbial diversity, cannot accurately quantify which species contributes to the metabolic power of Daqu, the size of its flavor compounds, and the flavor markers produced, as well as the underlying mechanisms.

The present study employed advanced biological and informatics analysis techniques. These included PacBio SMRT ITS sequencing technology, fungal genome *de novo* sequencing, and Headspace Solid-Phase Micro-extraction/Gas Chromatography–Mass Spectrometry (HS-SPME/GC–MS) analysis of volatile compounds. Additionally, the study examined the flavor markers produced by the three fungal species and their formation mechanisms. The investigation focused on the abundance of these fungi, their liquefaction, saccharification, fermentation, and esterification power, as well as their impact on fungal volatiles, simulated volatiles in solid fermentation cultures, and metabolic relationships. This research aids in the development of a comprehensive fungal flora indicator system for Daqu, which can help stabilize its quality and facilitate the transformation of Chinese Baijiu production from traditional methods to intelligent processes.

## 2 Materials and methods

### 2.1 Sources of Fengxiangxing Daqu and fungal samples

Five replicate batches of Fengxiangxing Daqu were obtained from Shaanxi Xifeng Liquor Company Limited in Liulin Town, Baoji City, Shaanxi Province. These were labeled as SC1, SC2, SC3, SC4, and SC5, respectively. The three fungal strains were isolated from Fengxiangxing Daqu and labeled MHR2, XFSa-P5, and XFTh-X1.

### 2.2 Analysis of fungal community composition in Fengxiangxing Daqu

Total DNA was extracted from Fengxiangxing Daqu using the Fungal Genome Extraction Kit (Qiagen, Venlo, Holland). PCR amplification of the full-length ITS region was performed using primers ITS1F (5′-CTTGGTCATTTAGAGGAAGTAA-3′) with a barcode and ITS4R (5′- TCCTCCGCTTATTGATA TGC-3′) (Souza et al., 2014). Library construction was performed using the SMRTbell Prep Kit 3.0, and sequencing was performed on the PacBio Sequel IIe System (Shanghai Meiji Biomedical Technology Co., Ltd.). Sequences were clustered into operational taxonomic units (OTUs), and chimeric sequences were excluded based on 97% similarity using Usearch 11 (Edgar Robert, 2013). The community composition of the samples was analyzed at both the genus and species levels.

### 2.3 Isolation and identification of fungal strains

#### 2.3.1 Isolation of fungal strains

(1) Isolation of strain MHR2: A sample was taken from the bright yellow region at the core of Daqu and examined under a stereoscopic microscope. The bright yellow ascocarp was selected and suspended in a sterile stroke-physiological saline solution to prepare a fungal suspension. This suspension was inoculated onto Bengal red agar plates and incubated at 30°C for 5 to 7 days. After colony growth, the bright yellow ascocarp was again selected and suspended in a sterile stroke-physiological saline solution to prepare a fungal suspension. This suspension was inoculated onto Bengal red agar plates and incubated at 30°C for 5 to 7 days to isolate strain MHR2. Representative colonies were transferred to slant agar in test tubes and cultured under the same conditions. The fully grown cultures were stored at 4°C. And morphological characterization of MHR2 using stereoscopic microscope, and scanning electron microscope.
(2) Isolation of strain XFSa-P5: White snowflake-like mycelia were scraped directly from the surface of Daqu under a stereoscopic microscope, suspended in sterile stroke-physiological saline solution, and used to prepare a fungal suspension. This suspension was streaked onto PDA plates and incubated at 30°C for 5–7 days. After colony growth, white mycelia were again suspended in a sterile stroke-physiological saline solution to prepare a fresh fungal suspension. This suspension was inoculated onto PDA plates and incubated at 30°C for another 5–7 days to isolate strain XFSa-P5. The strain was transferred to slant agar in test tubes and cultured under the same conditions. The fully grown cultures were stored at 4°C. And morphological characterization of XFSa-P5 using stereoscopic microscope, and scanning electron microscope.
(3) Isolation of strain XFTh-X1: A sample was taken from the light red or purple area in the core of Daqu and examined under a stereoscopic microscope. A cloud-like section with a red or purple hue was selected and suspended in a sterile stroke-physiological saline solution to prepare a fungal suspension. This suspension was inoculated onto PDA plates and incubated at 48°C for 5–7 days until well-developed colonies were observed. A purple-red cloud-like colony was then picked and resuspended in sterile stroke-physiological saline solution to prepare a second fungal suspension. This suspension was inoculated onto PDA plates and incubated at 48°C for 5–7 days to obtain strain XFTh-X1. The strain was transferred to slant agar in test tubes and cultured under the same conditions. The fully grown cultures were stored at 4°C. And morphological characterization of XFTh-X1 using stereoscopic microscope, and scanning electron microscope.

#### 2.3.2 ITS rRNA sequencing of the fungal strains

Genomic DNA was extracted from the fungal strains MHR2, XFSa-P5, and XFTh-X1 using the Fungal Genomic DNA Extraction Kit, following the instructions for the manufacturer. PCR amplification was performed using the primers ITS1F and ITS4R. A small aliquot of the PCR product was analyzed using 1% agarose gel electrophoresis. The PCR product containing the target fragment was sent to Shanghai Meiji Biomedical Science and Technology Co. Ltd. for sequencing. The assembled sequences were then uploaded to the BLAST online tool^1^ for comparison, and the homology with the reference strain was determined. ITS rRNA gene sequences with higher homology were selected, and a phylogenetic tree was constructed using MEGA 6.0 software.

#### 2.3.3 Whole genome sequencing and evolutionary analysis

Total DNA from the mycelia of MHR2, XFSa-P5, and XFTh-X1 was extracted using the Fungal Genome Extraction Kit. The purified genomic DNA was quantified, and high-quality DNA was used for library construction and subsequent sequencing. Paired-end sequencing (2 × 150 bp) was performed using the Illumina NovaSeq6000 platform. The sequencing data were processed for bioinformatics analysis, and the raw data from the downstream analysis were stored in FASTQ format. All analyses were performed on the cloud platform provided by Shanghai Majorbio Bio-pharm Technology Co., Ltd.^2^

### 2.4 Metabolic power assay of the fungal strains

For the simulated Daqu solid-state fermentation medium, barley, wheat, and peas were mixed in a 6:1:3 ratio, crushed, and thoroughly homogenized. The moisture content was adjusted to 30–40%, and the mixture was loaded at a rate of 200 g per 1,000 mL, then divided into 1,000 mL conical flasks. The fungal strains MHR2, XFSa-P5, and XFTh-X1 were inoculated into the simulated Daqu solid-state fermentation medium at an inoculum concentration of 5%. MHR2 and XFSa-P5 were incubated at 30°C for 10 days, while XFTh-X1 was incubated at 48°C for 10 days. After incubation, the metabolic activities of the strains were evaluated using the QBT4257-2011 general analytical method for winemaking (Ministry of Industry and Information Technology of the People’s Republic of China, 2012).

### 2.5 Morphological observations of the fungal strains

The strains MHR2, XFSa-P5, and XFTh-X1 were inoculated onto PDA medium plates. MHR2 and XFSa-P5 were cultured at 30°C, while XFTh-X1 was cultured at 48°C. The colony morphology of the three strains was observed by the naked eye after 5 d of incubation. The plates were then examined under a stereomicroscope for a more detailed observation of the colony morphology. At the same time, a small amount of the fungi on the plates was collected and placed under a scanning electron microscope to observe the fungal morphology at a higher resolution.

### 2.6 HS-SPME/GC–MS analysis

#### 2.6.1 Sample preparation

For mycelial samples, strains MHR2, XFSa-P5, and XFTh-X1 were inoculated onto PDA medium plates using line inoculation. MHR2 and XFSa-P5 were incubated at 30°C for 8 days, while XFTh-X1 was incubated at 48°C for 8 days for the same duration.

For the simulated Daqu solid-state fermentation samples, the strains MHR2, XFSa-P5, and XFTh-X1 were each inoculated into Daqu fermentation medium at an inoculation rate of 5%. MHR2 and XFSa-P5 cultures were incubated at 30°C for 8 days, whereas XFTh-X1 was incubated at 48°C for 8 days.

#### 2.6.2 Detection conditions

A 2.00 g sample from section 2.6.1 was placed into a headspace vial. Before loading, 10 μL of the internal standard 2-octanol (at a concentration of 50.0 μg/mL) was added to each vial. Volatile compounds were then extracted using solid-phase microextraction with an autosampler equipped with a DVB/C-WR/PDMS extraction head (Shimadazu, Tokyo, Japan). The sample was incubated at 250°C for 5 min. The heating procedure consisted of an initial hold at 45°C for 3 min, followed by a temperature increase to 230°C at a rate of 4°C/min, with a final hold at 230°C for 6 min. Helium (He) was used as the carrier gas at a flow rate of 3 mL/min, and the sample was injected in a spitless mode. The electron impact (EI) ionization source was used with an electron energy of −70 eV. The ion source and injection port temperatures were set to 210°C and 250°C, respectively. Mass spectrometry data were acquired in the 35.0–350.0 m/z range (Huang et al., 2023; Liu et al., 2022b). The data obtained from the GC–MS analysis were processed using GC–MS Solution 4.52 software. Qualitative analysis was conducted by comparing the mass spectra against the NIST 20.0 standard spectral library. Results with more than 80% similarity were used for further qualitative analysis. The relative contents of the components were calculated using the internal standard method. The relative contents of the compounds were calculated using the internal standard method. The formula was as follows: $C=\frac{A}{A_{i}}\times \frac{C_{i}\times V_{i}}{M}\times 10^{-3}$, where C is the content of each volatile compound (μg/kg); A is the peak area of each component; C_i_ is the mass concentration of the internal standard (μg/mL); V_i_ is the volume of the internal standard (μL); A_i_ is the peak area of the internal standard; and M is the mass of the sample (kg).

#### 2.6.3 Quantification of flavor markers

##### 2.6.3.1 Standard curves

The standard (0.0200 g ± 0.0001 g) was accurately weighed, dissolved in anhydrous ethanol, and diluted to 10 mL to prepare a stock solution, which was stored in a sealed container at 4°C. Subsequently, 25.00 μL of the stock solution (2 mg/mL) was accurately pipetted and diluted with anhydrous ethanol to 25 mL to create an intermediate solution, also stored at 4°C. From the intermediate solution (5 μg/mL), different working solutions were prepared by pipetting specific volumes, and diluting to the mark with distilled water. For each working solution, 1 mL was transferred into a vial, followed by the addition of 0.5 g NaCl and 20 μL of 2-octanol standard solution (50 μg/mL). The vials were tightly closed, mixed thoroughly, and analyzed using HS-SPME/GC–MS (Cheng et al., 2024). The concentrations of the standards were plotted on the horizontal coordinate, and the peak areas of the compounds in the working solution were plotted on the vertical coordinate to plot the standard curve (Cheng et al., 2024).

##### 2.6.3.2 Quantification of flavor markers

For the preparation of mycelial flavor marker samples, strains MHR2 and XFSa-P5 were inoculated on medium plates for mycelial culture using line inoculation and incubated at 30°C. Mycelium was scraped on the 0th, 3rd, 6th, and 9th days.

For flavor marker samples from simulated Daqu solid-state fermentation cultures, strains MHR2 and XFSa-P5 were inoculated into a simulated Daqu solid-state fermentation medium at a 5% inoculation rate. The culture was incubated at 30°C, and samples were collected on the 0th, 2nd, 4th, 6th, and 8th days.

For analysis, 2.0000 g ± 0.0001 g of each solid sample was weighed, and 4 mL of distilled water, 1.5 g of NaCl, and 20 μL of 2-octanol standard solution (50 μg/mL) were added. After sealing and mixing, the samples were analyzed using HS-SPME/GC–MS (Cheng et al., 2024). The flavor marker content was quantified based on the standard curve established in section 2.6.3.1.

#### 2.6.4 Relative aroma activity value

The relative aroma activity value (*r*OAV) was calculated based on the concentration of the main compounds and their corresponding olfactory threshold values. *r*OAV is the ratio of the mass concentration of each odor compound in water to its respective odor threshold value. The olfactory thresholds for the compounds were referenced from the Goodscents Company Odor website. The aroma profiles of the compounds were determined using the Goodscents Flavor and Fragrance Information System.

### 2.7 Statistical analysis

Data processing and graphical representations were performed using Excel 2019 and GraphPad Prism 8.0 software. Multifactor analysis of variance (ANOVA) was performed using PASW 18.0 software. The results were expressed as Mean ± standard deviation (SD), with statistical significance considered at *p* < 0.05, denoted by an asterisk (*).

## 3 Results

### 3.1 Abundance and metabolic power of the three fungi in Daqu

#### 3.1.1 Abundance of the three fungi in Daqu

**Figure 1:**
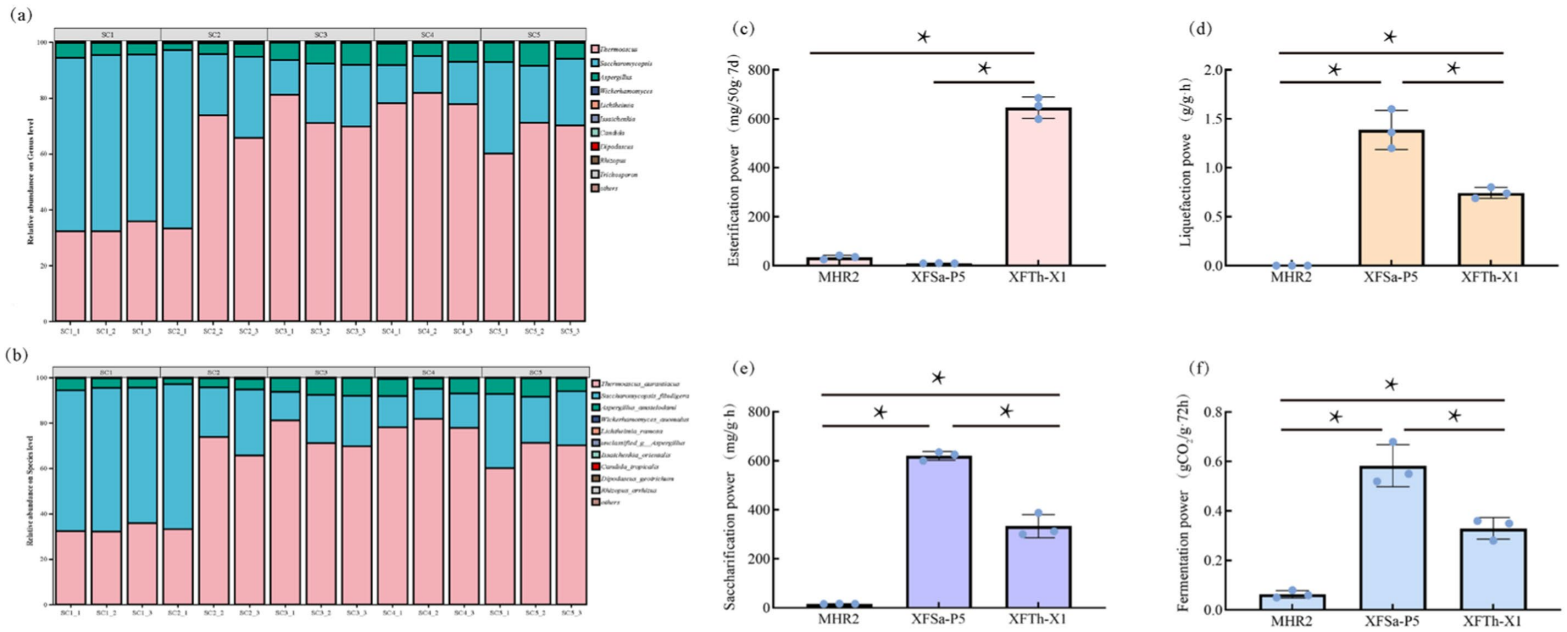
Fungal composition of Fengxiangxing Daqu at the genus level **(a)** and species level **(b)**, and the metabolic power of the fungal strains MHR2, XFSa-P5, and XFTh-X1 in simulated Daqu solid-state fermentation cultures (**c**: esterification power, **d**: liquefaction power, **e**: saccharification power, **f**: fermentation power). * denotes *p* < 0.05.

The collected Fengxiangxing Daqu samples were analyzed using PacBio full-length fungal sequencing, and the results are presented in Figures 1a,b. At the genus classification level, the three most abundant genera were *Thermoascus* (62.22%), *Saccharomycopsis* (31.69%), and *Aspergillus* (5.70%) (Figure 1a). At the species level, the three most abundant species were *Thermoascus aurantiacus* (62.22%), *Saccharomycopsis fibuligera* (31.69%), and *Aspergillus chevalieri* (5.70%), with three fungi in SC1-SC5 samples relative abundances of 99.7, 99.5, 99.8, 99.6 and 99.7%, respectively. These findings showed that these three fungi were dominant and stable in Daqu (Figure 1b). Furthermore, these fungi are commonly detected as dominant fungi in other types of Daqu (Yang et al., 2022; Hou et al., 2022; Jin et al., 2019).

#### 3.1.2 Metabolic power of the three fungi

The liquefaction, saccharification, fermentation, and esterification power of the three fungi in the simulated Daqu solid-state fermentation medium are shown in Figures 1c–f. The results showed that the liquefaction, saccharification, fermentation, and esterification power of MHR2 were comparatively low. In contrast, XFSa-P5 had the highest liquefaction and saccharification power, with values of 1.6 ± 0.16 g/g·h and 625.5 ± 14.68 mg/g·h, respectively. Additionally, its fermentation power was also significantly higher than that of MHR2 and XFTh-X1. The XFTh-X1 strain demonstrated a significantly higher esterification power than the other two strains, at 599.34 ± 35.54 mg/50 g·7d (*p* < 0.05).

These findings suggest that MHR2 had a lower effect on the metabolic power of the Daqu. In comparison, XFSa-P5 and XFTh-X1 were the primary contributors to the metabolic power, as reflected in the physicochemical indices of Fengxiangxing Daqu.

### 3.2 Identification of the three fungi

#### 3.2.1 Morphological characteristics of the three fungi

**Figure 2:**
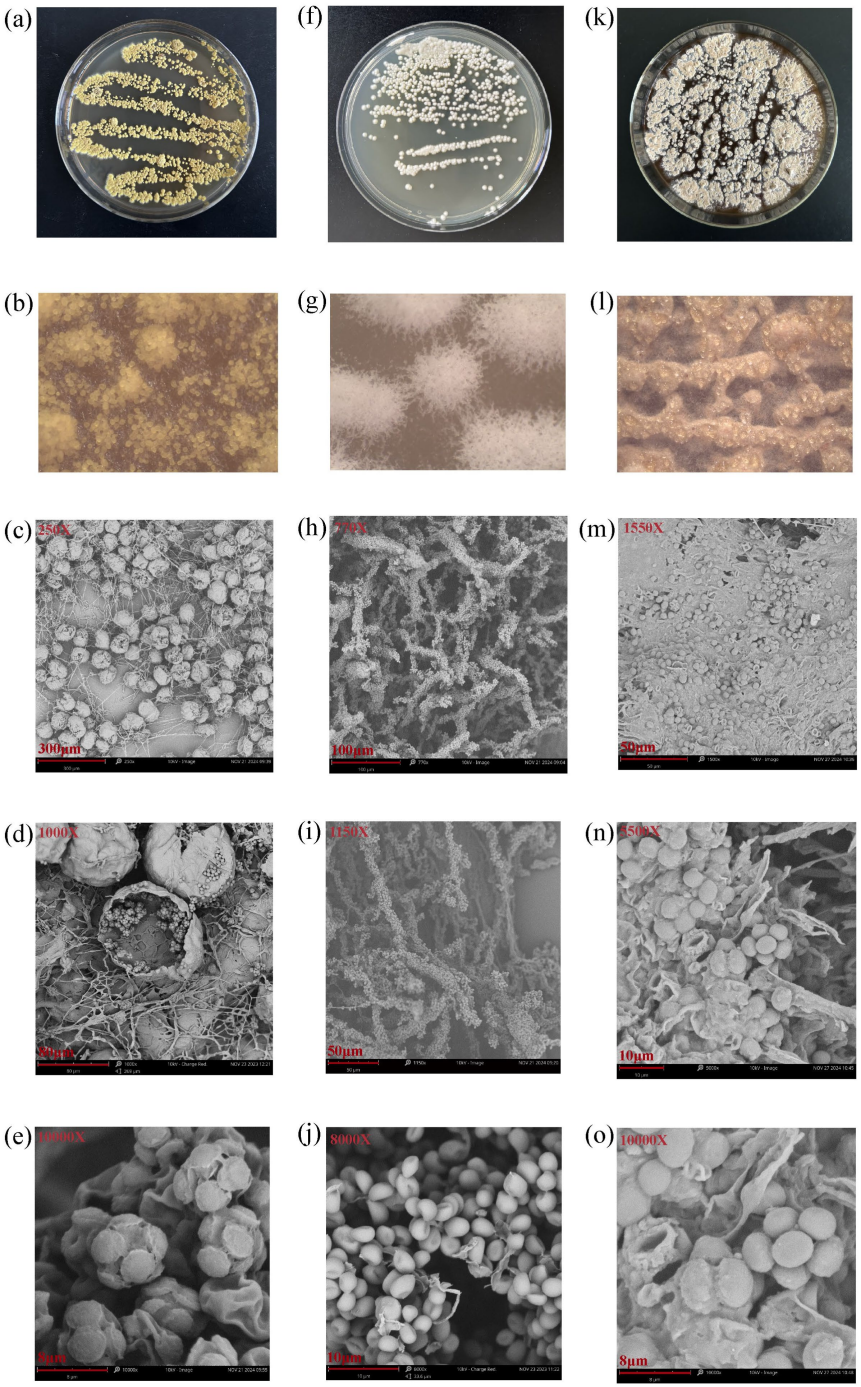
Colony morphology, stereomicroscopic observations, and scanning electron microscopy images of the three core fungi (**a–e**: MHR2; **f–j**: XFSa-P5; **k–o**: XFTh-X1).

The colony morphology of the three fungi on culture plates, the colony morphology under a stereomicroscope, and the mycelial morphology observed using a scanning electron microscope are shown in Figure 2. The colony and mycelial morphology of the MHR2 strain closely resembled the morphological characteristics of *Aspergillus chevalieri* (Chen et al., 2017). Similarly, the colony and mycelial morphology of the XFSa-P5 strain were consistent with the morphological characteristics of *Saccharomycopsis fibuligera* (Barnett et al., 2000). The colony and mycelial morphology of the XFTh-X1 strain were closer to that of *Thermoascus aurantiacus* (Wei et al., 1979; Zhong et al., 2024).

#### 3.2.2 ITS rRNA sequencing results and phylogenetic trees of the three fungi

**Figure 3:**
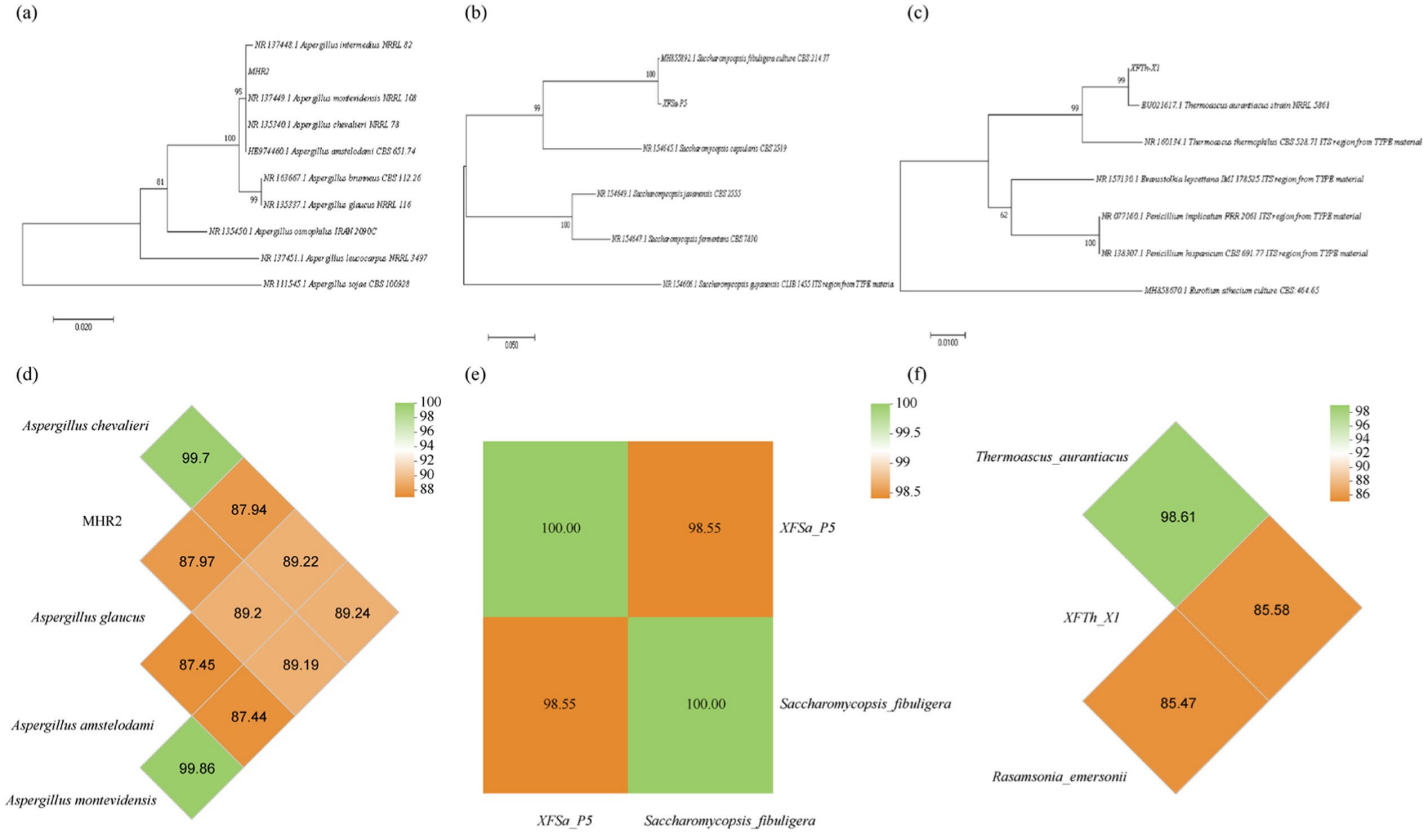
Phylogenetic tree and genome-wide average nucleotide identity (ANI) analysis of the three fungi based on ITS rRNA sequences using the neighbor-joining method (**a,d**: MHR2, **b,e**: XFSa-P5, **c,f**: XFTh-X1).

The ITS rRNA sequencing results for the three fungi were analyzed using BLAST sequence comparison and clustering analysis performed with MEGA 6.0 software. The MHR2 strain was grouped within the same phylogenetic branch as *Aspergillus chevalieri* MZ573106.1, *Aspergillus amstelodami* MN187972.1, and *Aspergillus montevidensis* NR137449.1 (Figure 3a). These results suggested that while the ITS rRNA phylogenetic tree classified MHR2 at the species level as *Aspergillus amstelodami*, *Aspergillus montevidensis*, or *Aspergillus chevalieri*, it did not allow definitive classification into a single species. The XFSa-P5 strain clustered within the same branch as *Saccharomycopsis fibuligera* MH855892.1 (Figure 3b). Therefore, this putative strain was designated as *Saccharomycopsis fibuligera* XFSa-P5. Similarly, the XFTh-X1 strain clustered within the same branch as *Thermoascus aurantiacus* JN676149.1 (Figure 3c) and was classified as *Thermoascus aurantiacus* XFTh-X1.

#### 3.2.3 Whole genome sequencing results and identification of the three fungi

The genome of strain MHR2 was 26,188,964 bp in length, with a G + C content of 49.38%. It contains 9,459 coding genes, accounting for 61.76% of the genome. Currently, seven complete genome sequences are available in the NCBI genome database. Strain M1 (GCA_016861735.1) served as the reference genome, with a genome size of 29,697,343 bp and a G + C content of 49.5%. Compared to the reference strain M1, the full-length genome of strain MHR2 was 26,188,964 bp, with a G + C content of 49.38% and 9,459 coding genes. These results confirm the accuracy and completeness of strain MHR2 genome sequencing.

The genome of strain XFSa-P5 was 15,791,323 bp in length, with a G + C content of 37.81%, and contained 5,788 coding genes, accounting for 64.36% of the genome. Strain KPH12 (GCA_001936155.1) served as the reference genome, with a genome size of 19,567,216 bp and a G + C content of 38%. Compared to the reference strain KPH12, the full-length genome of strain XFSa-P5 was 15,791,323 bp, with a G + C content of 37.81% and 5,788 coding genes. These results confirmed the accuracy and integrity of the genome sequencing of strain XFSa-P5.

The genome of strain XFTh-X1 had a total length of 28,114,151 bp, with a G + C content of 51.22%. It contained 6,027 coding genes, accounting for 58.06% of the genome. Strain Theau2 (GCA_042370945.1) served as the reference genome, with a genome size of 28,490,209 bp and a G + C content of 52%. Compared to the reference strain Theau2, the genome of strain XFTh-X1 was 28,114,151 bp long, with a G + C content of 51.22% G + C and 6,027 coding genes. These results preliminarily confirmed the accuracy and completeness of strain XFTh-X1 genome sequencing.

The OrthoANI algorithm was used to assess interspecies affinities at the genomic level. The whole-genome sequence of strain MHR2 was compared with the sequences of *Aspergillus amstelodami* GCA 027569315.1, *Aspergillus montevidensis* GCA 020826735.1, and *Aspergillus chevalieri* GCA 016861735.1 for average nucleotide identity (ANI) analysis (Figure 3d). The results showed that MHR2 had an ANI of 99.7% with *Aspergillus chevalieri*, greater than the species delineation threshold of 96%, while the ANI values with the other strains were below 90%. These findings indicated that MHR2 and *Aspergillus chevalieri* were the same species, consistent with morphological identification.

The whole genome sequence of strain XFSa-P5 was compared with that of *Saccharomycopsis fibuligera* (GCA 001936155.1). The ANI between XFSa-P5 and *Saccharomycopsis fibuligera* was 98.55%, also higher than the 96% threshold (Figure 3e). Therefore, XFSa-P5 was identified as the same species as *Saccharomycopsis fibuligera*.

The whole genome sequence of strain XFTh-X1 was compared with that of *Thermoascus aurantiacus* GCA 042370945.1. The ANI between XFTh-X1 and *Thermoascus aurantiacus* was 98.61%, greater than the species threshold (Figure 3f). Therefore, XFTh-X1 was identified as the same species as *Thermoascus aurantiacus*.

### 3.3 Differences in the volatile compounds of the three fungal mycelia

**Figure 4:**
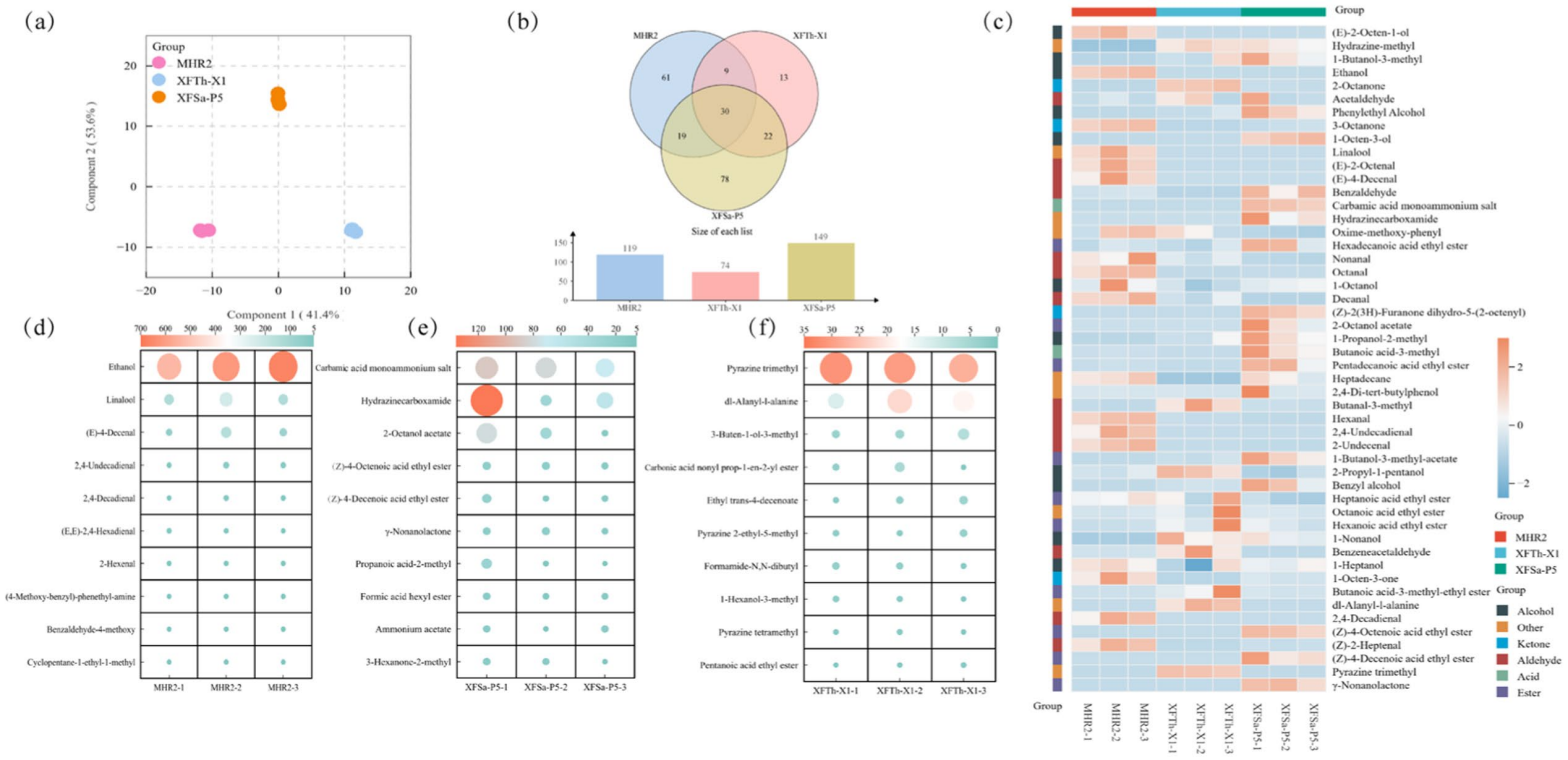
(O)PLS-DA plot of volatile compounds **(a)**, VENN plots **(b)**, distribution of the top 50 volatile compounds based on average relative contents in the mycelia of the three fungal strains **(c)**, and the top 10 unique volatile compounds based on average relative content in the mycelia of strains MHR2, XFSa-P5, and XFTh-X1 **(d,e,f)**.

The differences in their profiles were assessed using (O)PLS-DA. The corresponding (O)PLS-DA score plot is shown in Figure 4a. The first principal component (Comp 1) and second principal component (Comp 2) accounted for 95.0% of the total variation in the volatile compounds across the three fungal mycelium samples.

In total, 233 volatile compounds were detected in the three fungal mycelia, including 48 esters, 32 alcohols, 36 aldehydes, 28 ketones, 7 acids, and 82 other compounds. A comparative analysis of the types and relative contents of the volatile compounds showed that 119 compounds were detected in the MHR2 strain, 149 in the strain XFSa-P5, and 74 in the strain XFTh-X1. Among these, 30 volatile compounds were common to all three fungal strains (Figure 4b). The highest total content of volatile compounds in the mycelium of both MHR2 and XFSa-P5 strains was found in alcohol compounds, whereas the highest total content in the mycelium of XFTh-X1 was attributed to compounds from other classes.

Among the top 50 volatile compounds, 36 were identified in the MHR2 strain, 40 in the XFSa-P5 strain, and 30 in the XFTh-X1 strain. In the MHR2 sample, aldehydes, alcohols, and other compounds were predominant. In the XFSa-P5 sample, esters, alcohols, and aldehydes were predominant. For XFTh-X1, alcohols, esters, aldehydes, and other compounds were the predominant groups. These results demonstrate significant differences in the types and contents of the major volatile compounds across the fungal strains (Figure 4c).

The top 10 unique volatile compounds by average relative content in the mycelium of strain MHR2 included the highest concentrations of ethanol, linalool, and (E)-4-decenal (Figure 4d). Linalool, ranking second to ethanol, was predominantly present in the samples, with a concentration of 167.856 ± 44.4 μg/Kg. This result suggested that strain MHR2 has significant potential for linalool production. The total terpenoid compounds in MHR2 mycelium amounted to 172.528 ± 44.9 μg/Kg, significantly exceeding the ester content in the mycelium of the other two strains (*p* < 0.05). These findings suggested that strain MHR2 has potential for terpenoid production.

The volatile compounds unique to the mycelium samples of strain XFSa-P5 included carbamic acid monoammonium salt, hydrazinecarboxamide, and 2-octanol acetate, which were among the highest (Figure 4e). Additionally, (Z)-4-octenoic acid ethyl ester, (Z)-4-decenoic acid ethyl ester, and γ-nonanolactone were the most abundant ester compounds. The total ester content in the mycelium of strain XFSa-P5 was 304.936 ± 107.8 μg/Kg, significantly higher than that in the mycelium of the other two strains (*p* < 0.05). These results suggested that strain XFSa-P5 has a strong potential for the production of total ester compounds.

The volatile compounds unique to the mycelium of strain XFTh-X1 included pyrazine trimethyl, dl-alanyl-l-alanine, and 3-buten-1-ol-3-methyl, with pyrazine trimethyl being the most abundant (Figure 4f). Pyrazine trimethyl is known to contribute to baking and nutty aromas. Other characteristic compounds included pyrazine-2-ethyl-5-methyl, pyrazine tetramethyl, and pyrazine methyl. The total content of pyrazine compounds in the mycelium samples of strain XFTh-X1 was 39.002 ± 0.8 μg/Kg, significantly higher than their content in the mycelium of the other two strains (*p* < 0.05). These findings indicated that strain XFTh-X1 has a strong potential for pyrazine compound production.

The *r*OAV of the top 10 unique compounds in the three fungal mycelia was evaluated. As shown in Table 1, only linalool had an *r*OAV greater than 1 in strain MHR2. Linalool, known for its low sensory threshold, significantly influenced the aroma of the sample, contributing a fruity, nectar-like flavor and demonstrating high abundance. In strain XFSa-P5, 2-octanol acetate, γ-nonanolactone, formic acid hexyl ester, and 3-hexanone-2-methyl had *r*OAV greater than 1, measuring 138.428, 5.834, 6.188, and 1.380, respectively. These four compounds were the primary contributors to the aroma of the sample. Among these, γ-nonanolactone, characterized by its coconut milk and floral aromas, had the highest *r*OAV and the greatest effect on the aroma of the samples. In strain XFTh-X1, only pyrazine trimethyl had an *r*OAV greater than 1, with a value of 1.295.

**Table 1:** Relative odor activity value (*r*OAV) of the top 10 unique compounds based on average relative content in the mycelium from the three fungal strains.

Sample	Compound	Odor threshold (ug·Kg^−1^)	Odor description	*r*OAV
MHR2	Ethanol	950,000	Alcoholic aroma	0.001
	Linalool	6	Floral aroma, woody aroma	27.979
	(E)-4-Decenal	106.01	Refreshing aroma	0.898
	2,4-Undecadienal	/	Caramel aroma, spicy aroma	/
	2,4-Decadienal	70	Fresh aroma, citrus aroma	0.246
	(E,E)-2,4-Hexadienal	18	Fruity aroma, green aroma	0.664
	2-Hexenal	110	Fruity aroma, green aroma	0.090
	(4-Methoxy-benzyl)-phenethyl-amine	/	/	/
	Benzaldehyde-4-methoxy	300	Hawthorn aroma	0.026
	Cyclopentane-1-ethyl-1-methyl	/	/	/
XFSa-P5	Carbamic acid monoammonium salt	/	/	/
	Hydrazinecarboxamide	/	/	/
	2-Octanol acetate	0.3	Fruity aroma	138.428
	(Z)-4-Octenoic acid ethyl ester	/	Sweet aroma, fruity aroma	/
	(Z)-4-Decenoic acid ethyl ester	/	Wax aroma, pear aroma	/
	γ-Nonanolactone	2.5	Coconut milk aroma	5.833
	Propanoic acid-2-methyl	57.5	Fruity aroma, green aroma	0.229
	Formic acid hexyl ester	2	Sweet aroma, fruity aroma	6.1888
	Ammonium acetate	/	/	/
	3-Hexanone-2-methyl	8	Fruity aroma	1.380
XFTh-X1	DL-Alanyl-L-alanine	/	/	/
	3-Buten-1-ol-3-methyl	600	Fruity aroma, green aroma	0.009
	Pyrazine trimethyl	23	Roasted potato aroma, nutty aroma	1.295
	Carbonic acid nonyl prop-1-en-2-yl ester	/	/	/
	Ethyl trans-4-decenoate	70	Wax aroma, pear aroma	0.039
	Pyrazine 2-ethyl-5-methyl	100	Nutty aroma	0.022
	Formamide N,N-dibutyl	/	/	/
	1-Hexanol 3-methyl	200	Leafy green aroma	0.007
	Pyrazine tetramethyl	124	Fermented aroma, coffee aroma	0.006
	Pentanoic acid ethyl ester	1.5	Pineapple fruit aroma	0.445

“/” indicates the threshold beyond which the compound was not detected.

### 3.4 Differences in volatile compounds of the three fungal solid-state fermentation cultures

**Figure 5:**
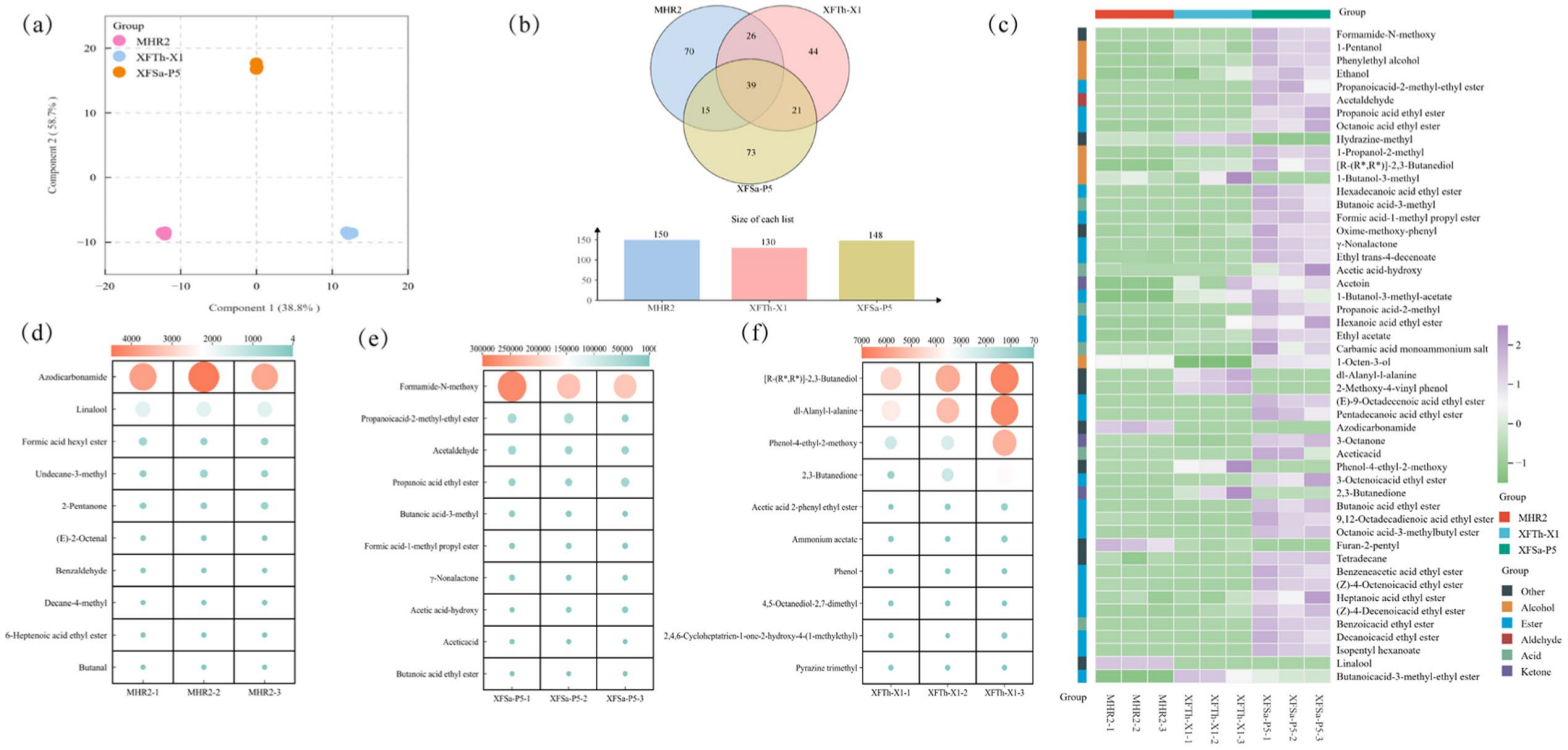
(O)PLS-DA plots of volatile compounds **(a)**, VENN plots **(b)**, distribution of the top 50 volatile compounds based on average relative content **(c)**, and distribution maps of the top 10 unique volatile compounds based on average relative content in the mycelium of strains MHR2, XFSa-P5, and XFTh-X1, which simulated Daqu solid-state fermentation cultures **(d,e,f)**.

The three strains were subjected to simulated Daqu solid-state fermentation, followed by a detailed analysis to investigate differences in their volatile compounds. The first principal component (Comp 1) and the second principal component (Comp 2) of the volatile compounds from the three fungal simulated Daqu solid-state fermentation cultures accounted for 97.5% of the total variation (Figure 5a).

A total of 288 volatile compounds, including 88 esters, 39 alcohols, 29 aldehydes, 38 ketones, 17 acids, and 77 other compounds, were detected in the three fungal simulated Daqu solid-state fermentation cultures. Comparative analysis of the types and relative contents of the volatile compounds in the cultures showed that 150, 148, and 130 compounds were detected in the samples from strains MHR2, XFSa-P5, and XFTh-X1, respectively. Among these, 39 volatile compounds were common across all three strains. On the other hand, there were 70 volatile compounds unique to strain MHR2, 73 unique to strain XFSa-P5, and 44 unique to strain XFTh-X1 (Figure 5b).

The top 50 volatile compounds included 31 species from strain MHR2, 42 from strain XFSa-P5, and 32 from strain XFTh-X1 (Figure 5c). Among these, esters and alcohols were predominant in the MHR2 samples. In contrast, esters, acids, and alcohols were predominant in the XFSa-P5 samples. In the XFTh-X1 samples, esters and alcohols were the predominant compounds. These results indicated significant differences in the types and contents of the major volatile compounds across the three fungal strains.

As shown in Figure 5d, among the top 10 volatile compounds unique to strain MHR2, azodicarbonamide, linalool, and formic acid hexyl ester were predominant. Azodicarbonamide was the most abundant compound in this sample, with a concentration of 4006.325 ± 312.3 μg/Kg, while linalool was present at 1721.962 ± 6.1 μg/Kg. Notably, linalool was the only compound that co-existed in both the mycelium and the simulated Daqu solid-state fermentation culture of strain MHR2. Its content in the simulated Daqu solid-state fermentation culture was significantly higher than that in the mycelium, suggesting that strain MHR2 has the potential to synthesize linalool.

As shown in Figure 5e, the volatile compounds unique to the simulated Daqu solid-state fermentation culture of strain XFSa-P5 included formamide-N-methoxy, propanoic acid-2-methyl ethyl ester, and acetaldehyde. Additionally, γ-nonanolactone was also detected in the simulated Daqu solid-state fermentation culture, with a concentration of 12405.527 ± 1528.7 μg/Kg, indicating that strain XFSa-P5 has the potential to synthesize γ-nonanolactone.

As shown in Figure 5f, the volatile compounds unique to the simulated Daqu solid-state fermentation culture samples of strain XFTh-X1 included [R-(R*, R*)]-2,3-butanediol, DL-Alanyl-L-alanine, and phenol-4-ethyl- 2-methoxy. Compared with the mycelium sample, one pyrazine compound, pyrazine trimethyl (226.793 ± 4.2 μg/Kg), was detected exclusively in the sample of strain XFTh-X1. This result indicated that strain XFTh-X1 has the potential to synthesize pyrazine compounds, which is consistent with the results obtained from the mycelium samples of strain XFTh-X1, as presented in section 2.3.

The *r*OAV of the top 10 unique compounds in the three fungal simulated Daqu solid-state fermentation cultures is presented in Table 2. The samples from strain MHR2 contained five compounds with *r*OAV greater than one, including linalool, formic acid hexyl ester, 2-pentanone, (E)-2-octenal, and butanal. In the strain XFSa-P5, eight compounds had *r*OAV greater than one, including γ-nonanolactone, propanoicacid-2-methyl-ethyl ester, acetaldehyde, propanoic acid ethyl ester, butanoic acid-3-methyl, formic acid-1-methyl propyl ester, acetic acid, and butanoic acid ethyl ester. The samples of strain XFTh-X1 contained four compounds with *r*OAV greater than one, including phenol-4-ethyl-2- methoxy, phenol, propanal 2-methyl, and pyrazine trimethyl.

**Table 2:** Relative odor activity value (*r*OAV) of the top 10 unique compounds based on average relative content in three fungal simulated Daqu solid-state fermentation cultures.

Sample	Compound	Odor threshold (ug·Kg^−1^)	Odor description	*r*OAV
MHR2	Linalool	6	Floral aroma, woody aroma	286.991
	Azodicarbonamide	/	/	/
	Formic acid hexyl ester	2	Sweet aroma, fruity aroma	244.453
	Undecane-3-methyl	/	/	/
	2-Pentanone	90	Woody aroma, banana flavor	4.272
	(E)-2-Octenal	3	Fat and meat aroma	46.286
	Benzaldehyde	300	Bitter almond flavor, malt flavor	0.368
	Decane-4-methyl	/	/	/
	6-Heptenoic acid ethyl ester	/	/	/
	Butanal	56.2	Malt flavor, chocolate flavor	1.194
XFSa-P5	γ-Nonanolactone	2.5	Coconut milk aroma	4962.194
	Formamide-N-methoxy	/	/	/
	Propanoicacid-2-methyl-ethyl ester	0.02	Fruity aroma, sweet aroma	217.507
	Acetaldehyde	167	Grass aroma, fruity aroma	210.542
	Propanoic acid ethyl ester	100	Fruit wine aroma	328.469
	Butanoic acid-3-methyl	0.069	Fruity aroma	21.451
	Formic acid-1-methyl propyl ester	1	Sweet aroma	13.597
	Acetic acid-hydroxy	/	/	/
	Aceticacid	13	Irritating odor	294.484
	Butanoic acid ethyl ester	0.053	Irritating odor	47.536
XFTh-X1	dl-Alanyl-l-alanine	/	/	/
	Phenol-4-ethyl-2-methoxy	13	Smoky, clove	259.974
	Ammonium acetate	/	/	/
	Phenol	3.9	Bad odor and burning smell	73.274
	4,5-Octanediol-2,7-dimethyl	/	/	/
	2,4,6-Cycloheptatrien-1-one-2-hydroxy-4-(1-methylethyl)	/	/	/
	8-Methylnonanoic acid ethyl ester	/	/	/
	Propanal 2-methyl	0.5	Fruit, rose fragrance, waxy smell	281.866
	Pyrazine trimethyl	23	Roasted potato, fried peanuts	5.507
	1-Propanol	1,200,000	Fragrant aroma	0.0001

“/” indicates the threshold beyond which the compound was not detected.

In summary, a significant correlation was observed between the volatile compounds in the mycelial samples and the simulated Daqu solid-state fermentation cultures of strains MHR2, XFSa-P5, and XFTh-X1. Strain MHR2 had a high content of terpenoid compounds, particularly with the potential to synthesize linalool, which contributed to floral and woody aromas. Strain XFSa-P5 had a high concentration of ester compounds, with the potential to synthesize γ-nonanolactone, which contributed to the coconut milk and sweet aromas. Strain XFTh -X1 had a higher concentration of pyrazine compounds, with the potential to synthesize pyrazine compounds, which contributed to the roasted and nutty aromas.

### 3.5 Quantitative results of flavor markers

#### 3.5.1 Quantification of linalool in strain MHR2

The results in sections 3.3 and 3.4 showed that linalool was identified as the primary flavor marker of strain MHR2. Accordingly, linalool was quantified in samples collected from the mycelium and simulated Daqu solid-state fermentation cultures during the cultivation of strain MHR2. The *r*OAV of linalool was subsequently calculated.

The retention time of linalool was determined to be 26.912 min. A linear regression equation was formulated based on the peak area ratio, yielding *y* = 1008.7x-0.11 (*r*^2^ = 0.9919). The results indicated that linalool content in the mycelium of strain MHR2 increased gradually during incubation, reaching 1.583 μg/g, with a corresponding increase in the *r*OAV to 263.891. In the simulated Daqu solid-state fermentation culture of strain MHR2, the linalool content increased from 0.280 μg/g to 1.511 μg/g during incubation. This increase followed a trend of increasing, reaching a maximum on the fourth day and then decreasing while maintaining a relatively high level in the later stages of incubation. The *r*OAV of linalool in this culture increased from 47.004 to 251.829. These findings indicated the significant contribution of strain MHR2 to the production of the linalool aroma.

#### 3.5.2 Quantification of lactones in strain XFSa-P5

The results in sections 3.3 and 3.4 showed that strain XFSa-P5 had the potential to produce lactone compounds. Based on this, seven potential lactone compounds were quantified, and their *r*OAV was calculated from both mycelial samples and solid-state fermentation cultures.

In the mycelium of strain XFSa-P5, γ-dodecalactone, γ-octalactone, γ-nonanolactone, and DL-mevalonic acid lactone were detected during incubation. Among these, γ-octalactone showed the highest level on day 9. In the simulated Daqu solid-state fermentation cultures of strain XFSa-P5, γ-dodecalactone, γ-octalactone, γ-nonanolactone, γ-caprolactone, and γ-decalactone were detected. The highest level of γ-nonanolactone was observed on day 8 of incubation. Comparative analysis revealed that lilac lactone was not detected in the mycelium or the solid-state fermentation culture. However, the levels of γ-nonanolactone, γ-dodecalactone, and γ-octalactone were higher in both mycelium and solid-state fermentation cultures, suggesting their significant impact on the overall aroma of the sample.

The aroma thresholds for γ-octalactone, γ-nonanolactone, γ-caprolactone, and γ-decalactone were obtained from the database. The *r*OAV of γ-octalactone and γ-nonanolactone in the mycelium of strain XFSa-P5 was greater than one. Specifically, the *r*OAV of γ-octalactone increased from 0 to 9.900, while that of γ-nonanolactone increased from 0 to 7.286. These findings indicated that lactone compounds were volatile components unique to strain XFSa-P5. Among them, γ-nonanolactone contributes the most to the aroma of the sample (Tables 3, 4).

**Table 3:** Changes in the content of seven lactone compounds in strain XFSa-P5.

Compound	Retention time	Standard curves	Content (μg·kg^−1^)	
			Mycelium	Solid cultures
γ-dodecalactone	47.283	*y* = 1.2886x-0.0777	<LOD −13.999	<LOD −0.137
γ-octalactone	46.388	*y* = 0.3012x-0.1102	<LOD −69.302	<LOD −81.156
γ-nonanolactone	38.337	*y* = 0.4813x + 0.006	<LOD −18.215	<LOD −2177.527
Lilac lactone	28.844	*y* = 0.0523x-0.0014	<LOD	<LOD
dl-Mevalonic acid lactone	49.947	*y* = 0.0118x + 0.012	<LOD −2.478	<LOD
γ-caprolactone	29.799	*y* = 0.0361x-0.0008	<LOD	9.652–63.658
γ-decalactone	35.090	*y* = 0.429x-0.0183	<LOD	1.046–6.147

The data in the table represent the mean ± standard deviation of three repeated experiments. LOD indicates the limit of detection.

**Table 4:** Changes in the relative odor activity value (*r*OAV) of seven lactone compounds in strain XFSa-P5.

Compound	Odor threshold (μg·kg^−1^)	*r*OAV	
		Mycelium	Solid cultures
γ-dodecalactone	/	/	/
γ-octalactone	7	0–9.900	8.711–12.165
γ-nonanolactone	2.5	0–7.286	0–871.011
Lilac lactone	/	/	/
dl-Mevalonic acid lactone	/	/	/
γ-caprolactone	90.6	/	0.106–0.702
γ-decalactone	1.1	/	0.951–5.589

“/” indicates the threshold beyond which the compound was not detected.

### 3.6 Linalool metabolic pathway in strain MHR2

Based on existing microbial linalool synthesis pathways (Zhou et al., 2020; Carrau et al., 2005) and metabolomic analysis of strain MHR2, two metabolic pathways for linalool were identified using KEGG. The first pathway involved the conversion of mevalonic acid (MVA) into isopentenyl diphosphate (IPP), a common precursor for terpene synthesis. This pathway, known as the MVA pathway, leads to the production of different terpenes, including linalool. The second pathway was the 1-deoxy-D-xylulose-5-phosphate/methylerythritol phosphate (DOXP/MEP) pathway. Unlike the MVA pathway, the DXP/MEP pathway differs in both the site of synthesis and the route used to produce IPP.

**Figure 6:**
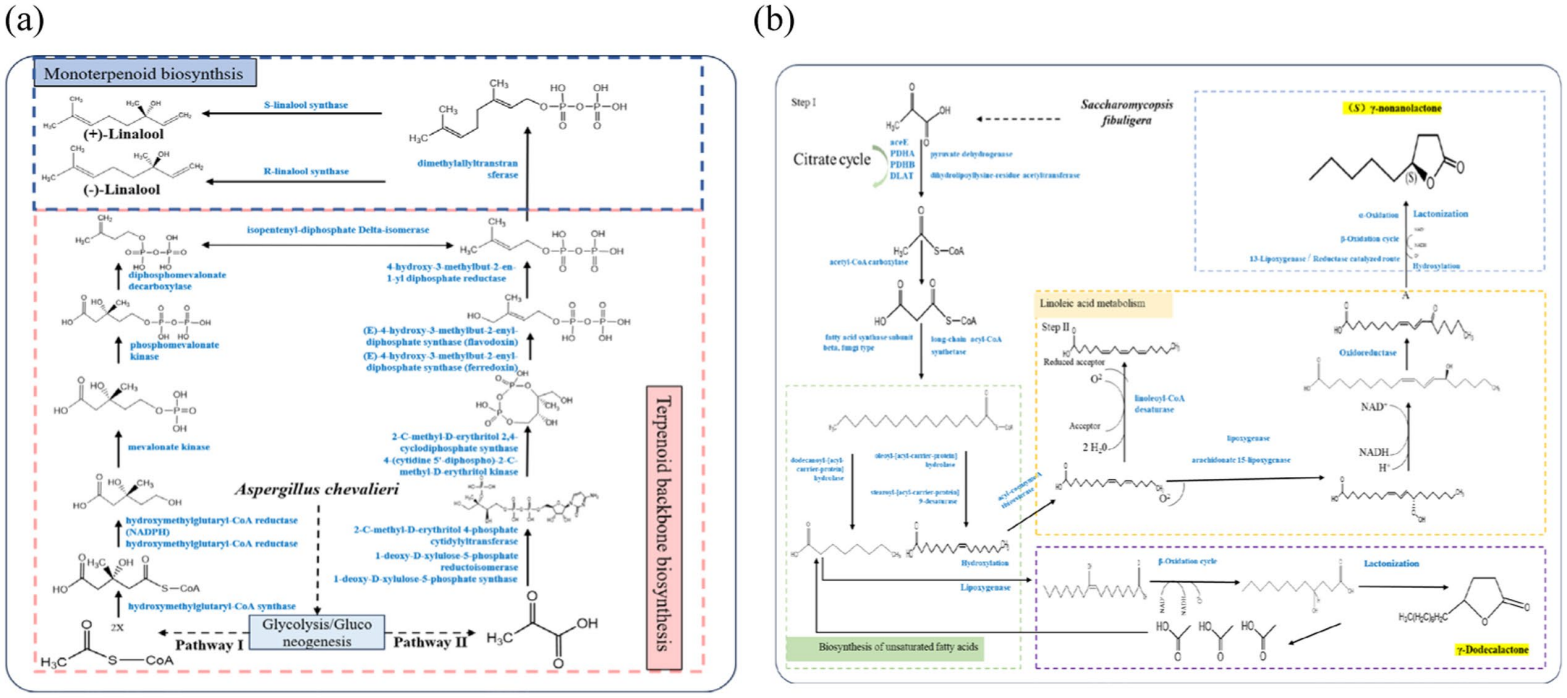
Possible metabolic pathways of linalool in strain MHR2 **(a)** and γ-nonanolactone in strain XFSa-P5 **(b)**. The blue font in the figure indicates the required enzyme or reaction process.

Several key enzyme-encoding genes involved in linalool production were identified based on whole-genome functional gene annotation. These included hydroxymethylglutaryl-CoA synthase (HMGCS), mevalonate kinase (MVK), phosphomevalonate kinase (PMVK), diphosphomevalonate decarboxylase (MVD), and farnesyl diphosphate synthase (FDPS). Specifically, strain MHR2 encoded one HMGCS, two MVK, one PMVK, one MVD and five FDPS genes. As shown in Figure 6a, strain MHR2 entered the MVA pathway to synthesize MVA from acetoacetyl-CoA and 3-hydroxy-3-methyl-glutaryl-CoA (HMG-CoA) in the presence of HMGCS and MVK. IPP was formed through subsequent phosphorylation and decarboxylation reactions. IPP was then catalyzed by FDPS to produce geranylpyrophosphate (GPP), a C10 backbone molecule that serves as a direct precursor for terpenoids. Finally, GPP enters the terpenoid skeleton biosynthetic pathway. In the presence of (3S)-linalool synthase, GPP is converted into (+)-linalool, whereas in the presence of (3R)-linalool synthase, it forms (−)-linalool. However, genome annotation revealed that strain MHR2 does not encode enzymes related to the DOXP/MEP pathway, suggesting that linalool synthesis occurs exclusively through the MVA pathway in this strain.

### 3.7 The γ-nonanolactone metabolic pathway of strain XFSa-P5

The biosynthesis of γ-nonanolactone involves key processes such as fatty acid hydroxylation, β-oxidation, lactonization, reduction of unsaturated lactones, Baeyer-Villiger oxidation of cyclic ketones, and the formation of macrolides through a, *ω*-oxidation of fatty acids to a, ω-dicarboxylic acids, followed by lactonization (Romero-Guido et al., 2011). Functional gene annotation of the whole genome indicated that strain XFSa-P5 encoded ten ACSL genes, three FAS1 genes, and two ACOT genes.

A possible metabolic pathway for γ-nonanolactone production by strain XFSa-P5 is shown in Figure 6b. Strain XFSa-P5 produced palmitic acid under the catalysis of FAS1. Palmitic acid was then catalyzed by ACSL to form palmitoyl coenzyme A, which underwent a series of elongation and desaturation reactions to yield linoleoyl-CoA. Linoleoyl-CoA was converted to linolic acid through the action of enzymes such as ACOT. Linolic acid was subsequently hydroxylated to form (9Z,11E)-(13S)-13-hydroperoxyoctadecane. Hydroperoxyoctadeca-9,11-dienoic acid is a key precursor compound for (S)-γ-nonanolactone synthesis. In the pre-metabolomics assay of strain XFSa-P5 mycelium, non-targeted metabolomics indicated a high abundance of this compound. Finally, (S)-γ-nonanolactone was synthesized through several cycles of β-oxidation followed by lactonization, constituting the primary pathway for γ-nonanolactone synthesis in strain XFSa-P5.

Meanwhile, strain XFSa-P5 synthesized γ-decalactone through a multi-step process. First, hydroxy fatty acids, non-hydroxy fatty acids, and fatty acid esters were used as substrates. These substrates underwent β-oxidation to generate the precursor compound 4-hydroxydecanoic acid. Finally, 4-hydroxydecanoic acid was converted into γ-dodecalactone through hydroxylation and carboxyl hydroxyl lactonization reactions (Figure 6b).

## 4 Discussion

In previous studies, *Aspergillus chevalieri*, *Saccharomycopsis fibuligera*, and *Thermoascus aurantiacus* were identified as the dominant microbial species in Fengxiangxing Daqu (Cao et al., 2024a; Chu et al., 2024)*. Thermoascus aurantiacus* showed significant positive correlations with the moisture and residual starch content of Daqu, as well as with compounds such as alcohols, aromatic compounds, phenols, and aldehydes (Cao et al., 2024a). Similarly, *Saccharomycopsis fibuligera* showed significant positive correlations with moisture, residual starch content, and acidity of Daqu and positively correlated with the production of acids, esters, and hydrocarbons (Cao et al., 2024a). Additionally, *Aspergillus chevalieri* was positively correlated with acidity and saccharification power in Daqu and was positively correlated with the production of acids, esters, terpenes, and hydrocarbons (Cao et al., 2024a; Cao et al., 2024b). Comparing the correlation between the dominant fungi in other Daqu types and their metabolic power showed that the dominant fungus *Thermoascus* in high-temperature Daqu exhibited strong esterification power, positively correlated with saccharification and liquefaction power. Similarly, the dominant fungus *Saccharomycopsis* in Daqu showed a highly significant positive correlation with the fermentation power of the samples (Kang et al., 2022), consistent with the results of this study. However, the abundance of *Aspergillus* positively correlated with the esterification power of samples in strong-flavored Daqu (Wang et al., 2022). These findings suggested that the results of the correlation analysis between the microbial flora and the metabolic power of Daqu, based on diversity analysis, are affected by the type of Daqu and subject to variability.

Therefore, these three fungi are pivotal in shaping the sensory quality, physicochemical properties, and flavor compounds of Fengxiangxing Daqu, playing a crucial role in the controlled enhancement of both Daqu and baijiu quality.

The XFTh-X1 strain positively influenced the esterification capacity of Daqu. The HS-SPME/GC–MS and OAV analyses demonstrated its potential for pyrazine compound production. As a thermophilic fungus, XFTh-X1 thrived in the high-temperature environment of Daqu fermentation. The heat-resistant hydrolases produced by the strain under these conditions enhanced the Maillard reaction, increasing the formation of pyrazine compounds (Deng et al., 2020). Studies have shown that the contents of 2,6-dimethylpyrazine, 2,3,5-trimethylpyrazine, 2-butyl-3,5-dimethylpyrazine, 3, 6-dimethyl-pyrazine, tetramethylpyrazine and methylpyrazine in high-temperature Daqu were higher than those in medium-high temperature, medium-temperature and low-temperature Daqu samples (Deng et al., 2023). The contents of *Thermoascus* and *Thermomyces* were positively correlated with pyrazines in high-temperature Daqu, which played an important role in the formation of soy sauce flavor of high-temperature Daqu (Deng et al., 2020).

The XFSa-P5 strain was capable of producing a wide range of lactone compounds. Through HS-SPME/GC–MS and OAV measurements, five lactone compounds, including γ-nonanolactone, were identified among the volatile flavor compounds produced by *Saccharomycopsis fibuligera*. γ-Nonanolactone is a compound with a complex aroma, including cream and caramel. γ-Decalactone imparted a fruity and peach-like aroma when diluted. These lactone compounds were abundant and contributed to the aroma of XFSa-P5 samples. Whole-genome sequencing and analysis further revealed the presence of three key genes associated with linolenic acid biosynthesis in *Saccharomycopsis fibuligera*. Linolenic acid is a precursor in the biosynthesis of γ-nonanolactone. The identification of these genes has revealed the metabolic pathway for γ-nonanolactone production in *Saccharomycopsis fibuligera*. This finding offers a theoretical basis for future strategies to enhance γ-nonanolactone production through metabolic pathway regulation, similar to the mechanism reported for the production of lactone compounds from fatty acids in yeast. Lactone compounds are widely distributed in different products, including baijiu, yellow wine, and whisky. In a related study, Su et al. (2020) reported that ester compound content increased by 11.8% during *Saccharomyces cerevisiae* fermentation compared to traditional fermentation agents. Additionally, *Saccharomycopsis fibuligera* produced various other flavor compounds, including esters, alcohols, organic acids, aldehydes, and ketones, contributing to an enhanced flavor experience in the final product (Su et al., 2020). Ge et al. (2021) reported that wine samples fermented with *Saccharomycopsis crataegensis* exhibited the highest total aroma activity and the richest diversity of aroma compounds, containing lactones (δ-decalactone, γ-nonanolactone, and β-nonanolactone) and the most abundant aroma compounds. Therefore, the production of lactone compounds, such as γ-nonanolactone, by strain XFSa-P5, as the dominant fungus in Fengxiangxing Daqu, plays a significant role in enhancing the flavor of wine.

The results of HS-SPME/GC–MS and *r*OAV analysis of the MHR2 strain showed that the fungus produced linalool, a terpene compound with a strong greenish-sweet aroma that resembles rosewood, along with floral notes of lilac, lily of the valley, and a blend of woody and fruity aroma profiles (Liu et al., 2025). Terpenes in baijiu are highly known for their aroma and flavoring properties and are considered essential functional compounds in the beverage. In recent years, research on terpenoids in baijiu has expanded significantly, with three primary sources identified: (1) terpenoids were introduced to baijiu through the raw materials used in brewing; (2) they were synthesized by microorganisms either *de novo* or via precursor compounds; and (3) they formed as a result of chemical reactions during the baijiu fermentation process (Feng et al., 2023). Terpene compounds were distributed across raw materials, Daqu, Zaopei, and finished wine during the production processes of Jiangxiangxing and Nongxiangxing baijiu, with high concentration observed during the fermentation of Daqu and the finished product. The highest content of total terpene compounds was found in Jiangxiangxing Daqu, at 2088.68 μg/kg (Zhao et al., 2024), followed by Nongxiangxing Daqu, which contained 2020.32 μg/kg (Feng et al., 2023). Although terpenoid compounds are a significant component of Chinese baijiu, particularly in Jiangxiangxing and Nongxiangxing varieties, there is a lack of specific research on the types, contents, and sources of terpenoids in Fengxiangxing baijiu. Linalool, a terpene compound, was investigated in this study. In simulated Daqu fermentation using *Aspergillus chevalieri*, the highest yield of linalool was 1.511 μg/g. A genome-wide analysis of the MHR2 strain using Maker2 software identified ten key genes involved in monoterpene synthesis, enabling the development of an initial metabolic pathway for linalool production. The identification of these genes sheds light on the molecular mechanisms underlying linalool biosynthesis in *Aspergillus chevalieri*, opening the possibility of enhancing flavor compound production through genetic engineering.

## 5 Conclusion

The findings identified *Aspergillus chevalieri*, *Saccharomycopsis fibuligera*, and *Thermoascus aurantiacus* as the core functional fungi in Fengxiangxing Daqu. It was demonstrated that *Aspergillus chevalieri* has low metabolic power in Daqu but has a strong capacity for producing chain terpene alcohol flavor compounds, with linalool as its flavor marker. *Saccharomycopsis fibuligera* was the main contributor to liquefaction power in Daqu, with additional fermentation and esterification powers, and is a significant producer of lactones, including the flavor marker γ-nonanolactone. *Thermoascus aurantiacus* was identified as the primary contributor to esterification power in Daqu while also exhibiting liquefaction, saccharification, and fermentation powers, with pyrazine identified as its flavor marker. These results revealed the distinct contributions of the three fungi to the physicochemical properties and flavor profiles of Daqu, as well as the formation mechanisms of the flavor markers linalool and γ-nonanolactone. Additionally, it supports the development of a comprehensive fungal flora indicator for Daqu, contributing to quality stabilization and facilitating the transformation of Chinese baijiu production from traditional methods to intelligent systems.

## Data Availability

The datasets presented in this study can be found in online repositories. The names of the repository/repositories and accession number(s) can be found here: https://www.ncbi.nlm.nih.gov/, PQ851626, PQ851635 and PQ851641; https://www.ncbi.nlm.nih.gov/genbank/ JBKJAG000000000, JBKKHO000000000 and JBKJAH000000000.

## Footnotes

^1^ https://blast.ncbi.nlm.nih.gov/Blast.cgi

^2^ cloud.majorbio.com
